# PTPRC functions as a prognosis biomarker in the tumor microenvironment of cutaneous melanoma

**DOI:** 10.1038/s41598-023-46794-6

**Published:** 2023-11-23

**Authors:** Xuemei Li, Zhanghui Yue, Dan Wang, Lu Zhou

**Affiliations:** Department of Dermatology, The Third Xiangya Hospital, Central South University, Changsha, Hunan Province 410000 People’s Republic of China

**Keywords:** Cancer, Immunology, Diseases

## Abstract

Cutaneous melanoma is one of the most malignant types of skin cancer, with an extremely poor prognosis. Immune cells infiltrated in the tumor microenvironment (TME) affects melanoma initiation, progression, prognosis and immunotherapy strategies in melanoma. The potential utility of TME-related genes as a prognostic model for melanoma and as a predictor of immunotherapeutic response merits further exploration. In this study, we determined that an immune-related gene, protein tyrosine phosphatase receptor type C (PTPRC), was positively correlated with the positive prognosis of melanoma patients. Integration of this gene with TNM classification created a predictive model that showed better performance in determining overall survival than others. PTPRC expression was positively correlated with the levels of immune checkpoint molecules, and PTPRC knockdown significantly enhanced the migration, invasion, and proliferation of melanoma cells. Finally, immunohistochemical results from HPA and Real-time quantitative PCR of clinical tissues confirmed that PTPRC expression was higher in melanoma than in normal skin. In conclusion, PTPRC served as a potential predictor of survival and response to immunotherapy in melanoma patients. The risk model combining the PTPRC and TNM classifications holds the potential to be a promising tool for prognostic prediction of cutaneous melanoma. This will help in the effective clinical management of melanoma patients.

## Introduction

Melanoma is a highly aggressive skin cancer originating from melanocytes, leads to approximately 55,500 deaths per year^1^. The etiology of malignant melanoma has not been fully understood. It is generally considered to be multifaceted, with race and genetics, trauma and irritation, sunlight, immunity, and other factors all likely to be involved^2^. Targeted therapies and immunotherapy have emerged over the past decade or so, improving overall survival (OS) for melanoma, particularly in immunotherapy. Immune checkpoint inhibitors such as anti-CTLA-4 monoclonal antibodies (Ipilimumab), anti-PD-1 monoclonal antibodies (Navulizumab and Pabrolizumab), and LAG-3 blocking antibodies (Relizumab) inhibit the reactivity of the corresponding immune checkpoint proteins and have proven to be clinically efficacious^3–6^. However, a subset of patients remained resistant to this immunotherapy, and some even experience autoimmune adverse effects^7,8^. Therefore, it is clinically meaningful to explore potential biomarkers to evaluate prognosis and assess whether patients would benefit from immunotherapy.

The tumor microenvironment (TME) is the soil on which tumorigenesis and development depend^9^. The two most important members in the TME infiltrating are mesenchymal cells and immune cells, playing an important role in tumor biology^10^. There is growing evidence that cancer cells can activate different immune pathways that lead to immunosuppressive functions and determine the immune microenvironment of tumors^11^. The association between robust lymphocytic infiltration and better patient survival has been well documented inovarian, head and neck, breast, uroepithelial, colorectal, lung, hepatocellular carcinoma, esophageal cancer, and melanoma^12^. Estimation of Stromal and Immune cells in Malignant Tumor tissues using Expression data (ESTIMATE) is a tool that uses gene expression data to predict tumor purity, and the presence of infiltrating stromal/immune cells in tumor tissue. The ESTIMATE algorithm is based on a single-sample genomic enrichment analysis and produces three scores: Stromal score (captures the presence of stroma in the tumor tissue); Immune score (represents the presence of immune cells in the tumor tissue), and Estimate score (infers tumor purity). Several studies have established that these scores correspond with clinicopathological aspects and chemotherapeutic treatment resistance in diverse tumor types and are thought to be valuable as prognostic indicators for patients^13–15^. In addition, TNM staging is valuable for cancer staging, prognosis determination, and treatment option selection, but it does not allow for accurate patient assessment from an immunological perspective^16^. Therefore, combining TNM classification with tumor microenvironment-related markers may be more useful for predicting the heterogeneous clinical behavior and prognosis of melanoma patients.

Herein, we explored the differentially expressed immune score-based genes related to the prognosis of melanoma, which resulted in the identification of one immune-related genetic biomarker that can be used to guide the prediction of prognosis and response to immunotherapy. Then, the function enrichment and immune landscape related to the prediction model were investigated. Next, we explored at how suppressing biomarker’ mRNA expression effected melanoma cell migration, invasion, and proliferation. Finally, we validated the biomarker protein levels and mRNA expression levels in the Human Protein Atlas (HPA) database and clinical specimens. In conclusion, these data suggest that the biomarker identified in this study, combined with the TNM staging of tumors, may be practical predictors of heterogeneous clinical behavior and prognosis in melanoma patients. It may provide a basis for future clinical treatment, especially immunotherapy.

## Results

### ImmuneScore highly related to the prognosis of melanoma patients

**Figure 1:**
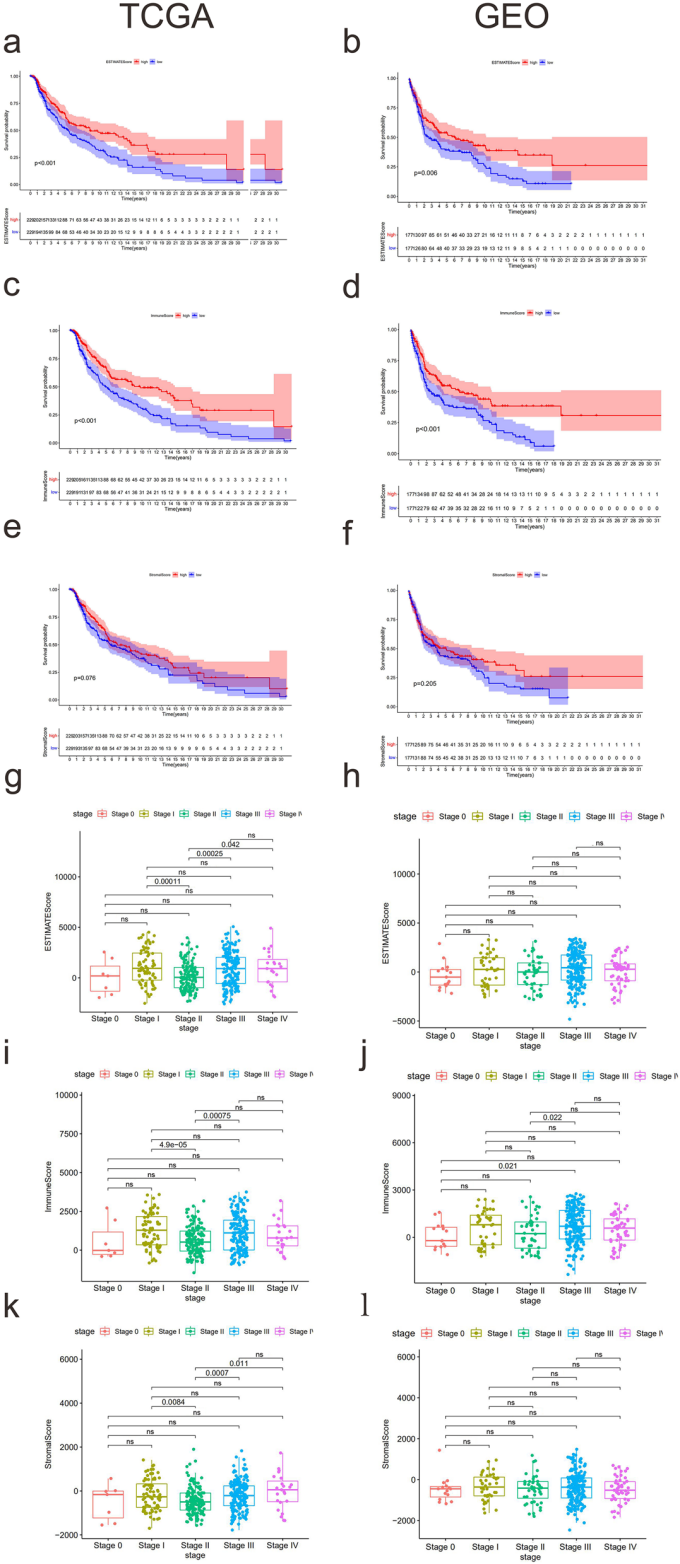
TME correlates with clinical traits and prognosis in patients with cutaneous melanoma. (**a**,**c**,**e**) Kaplan–Meier survival analysis of ESTIMATEScore, ImmuneScore, and StromalScore in TCGA. (**b**,**d**,**f**) Kaplan–Meier survival analysis of ESTIMATEScore, ImmuneScore, and StromalScore in GEO. (**g**,**i**,**k**) The boxplot of ESTIMATEScore, ImmuneScore, and StromalScore of cutaneous melanoma patients in different stages in TCGA. (**h**,**j**,**l**) The boxplot of ESTIMATEScore, ImmuneScore, and StromalScore of cutaneous melanoma patients in different stages in GEO.

A total of 470 tumor samples in the TCGA database and 332 tumor samples in the GEO datasets were acquired. The ESTIMATE algorithm was used to calculate ImmuneScore, StromalScore, and ESTIMATEScore for each sample. Based on the median of each score, the samples were divided into high or low-score groups, and Kaplan–Meier survival analysis was performed for each of the three scores. The results revealed that melanoma patients with higher ESTIMATEScore (*P* < 0.001; Fig. 1a) and ImmuneScore (*P* < 0.001; Fig. 1c) had significantly longer OS. However, there was no significant association between StromalScore and OS (*P* = 0.076; Fig. 1e). Similar results were observed in the GEO dataset (Fig. 1b,d,f). Also, the associations between the three scores and clinical traits were investigated using clinical data extracted from TCGA and GEO. We found that the three scores were notably declined in T4 (Supplementary material Fig S1a–c). Interestingly, all the three scores decreased at stage II and then increased at stage III as the tumor stage progressed in TCGA (Fig. 1g,i,k). While, in the GEO dataset, ImmuneScore decreased in stage II without significant difference, then increased at stage III with significant difference (Fig. 1j), the other two scores were not significantly associated with stage (Fig. 1h,l). In TCGA, all three scores were higher for patients in the lower age group (Supplementary material Fig S1d–f).

### Identification of differentially expressed genes (DEGs) related to immunity

**Figure 2:**
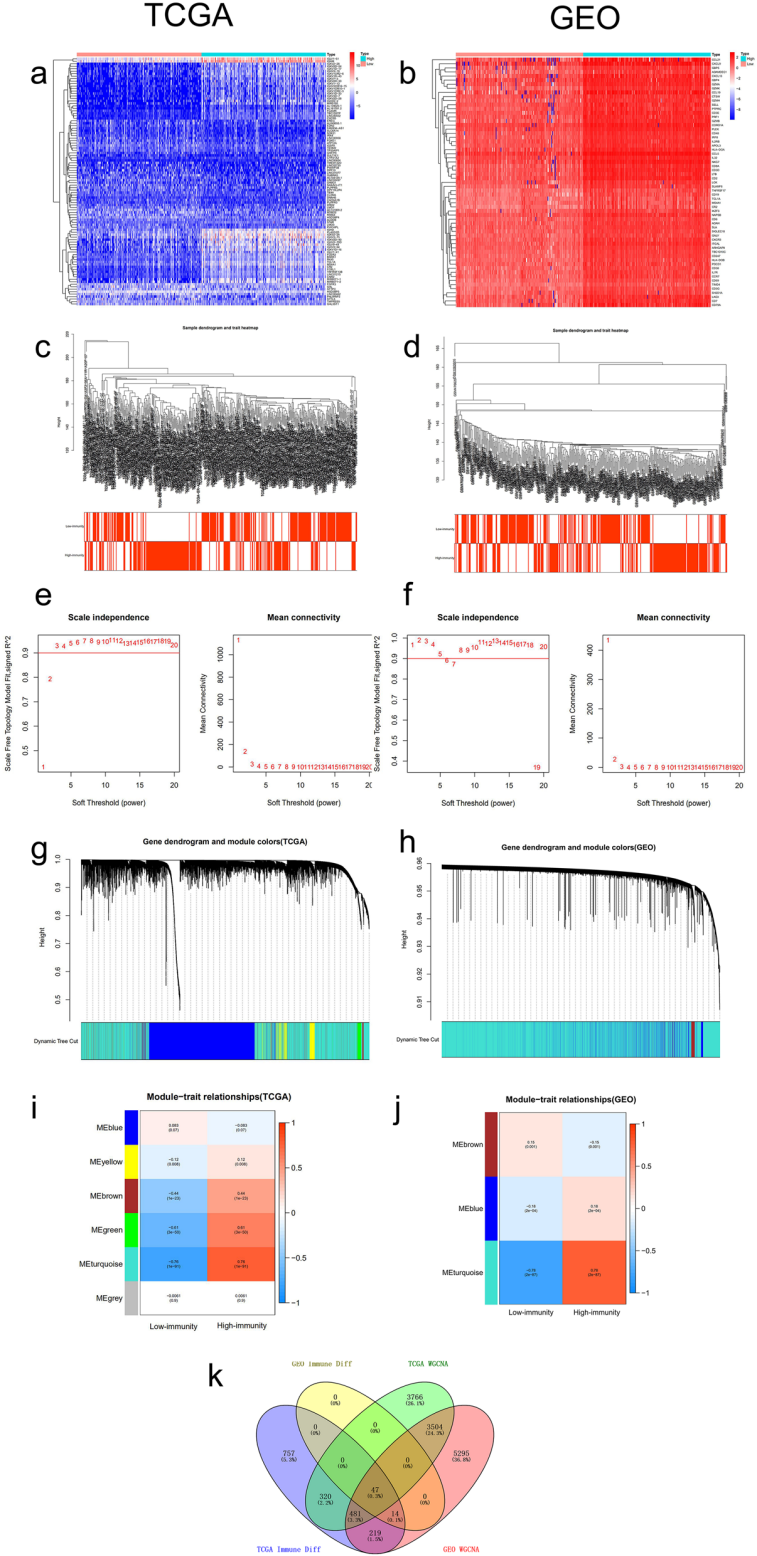
Identification of immune-related genes. (**a**,**b**) The heatmap of DEGs based on ImmuneScore grouping in TCGA and GEO. (**c**,**d**) Cluster dendrogram of high- and low-immunity melanoma samples from TCGA and GEO. (**e**,**f**) Soft-thresholding power in TCGA (*P* = 3) and GEO (*P* = 1). (**g**,**h**) Clustering dendrogram of identified gene modules in TCGA and GEO. (**i**,**j**) The heatmaps of identified functional gene modules and corresponding clinical traits in TCGA and GEO. (**k**) Venn plots of the intersection of genes related to high-immunity and previously filtered genes in TCGA and GEO.

Since the immune microenvironment plays an important role in melanoma behavior and disease prognosis, and the previous results indicate a strong relationship between ImmuneScore and clinical OS. To further explore the role of immune-related genes in melanoma, we divided the patients to high- and low- score groups according to their ImmuneScore and then identified DEGs. The analysis found 1684 significantly upregulated genes and 154 significantly downregulated genes in TCGA, and 61 significantly upregulated genes in GEO (Fig. 2a,b).

Weighted gene co-expression network analysis (WGCNA) was conducted to identify the critical gene modules closely associated with the high-immunity status of melanoma (Fig. 2c–f). In TCGA, five functional gene modules were identified (Fig. 2g,i), while in GEO, three functional gene modules were found (Fig. 2h,j). The turquoise module was the closest correlated to high-immunity status and was chosen for further analysis. Using Venn plots, we visualized the intersection of genes in the turquoise module and previously filtered genes (Fig. 2k). We found a total of 47 DEGs from the Venn plots, all of which were upregulated in the high-immunity group.

### PTPRC associated with OS in melanoma patients

To investigate the relationship between the immune-related DEGs and OS in melanoma patients, survival analysis on the 47 genes previously mentioned was conducted. We found that all 47 genes were associated with OS (*P* < 0.05) (Supplementary material Table S2).

**Figure 3:**
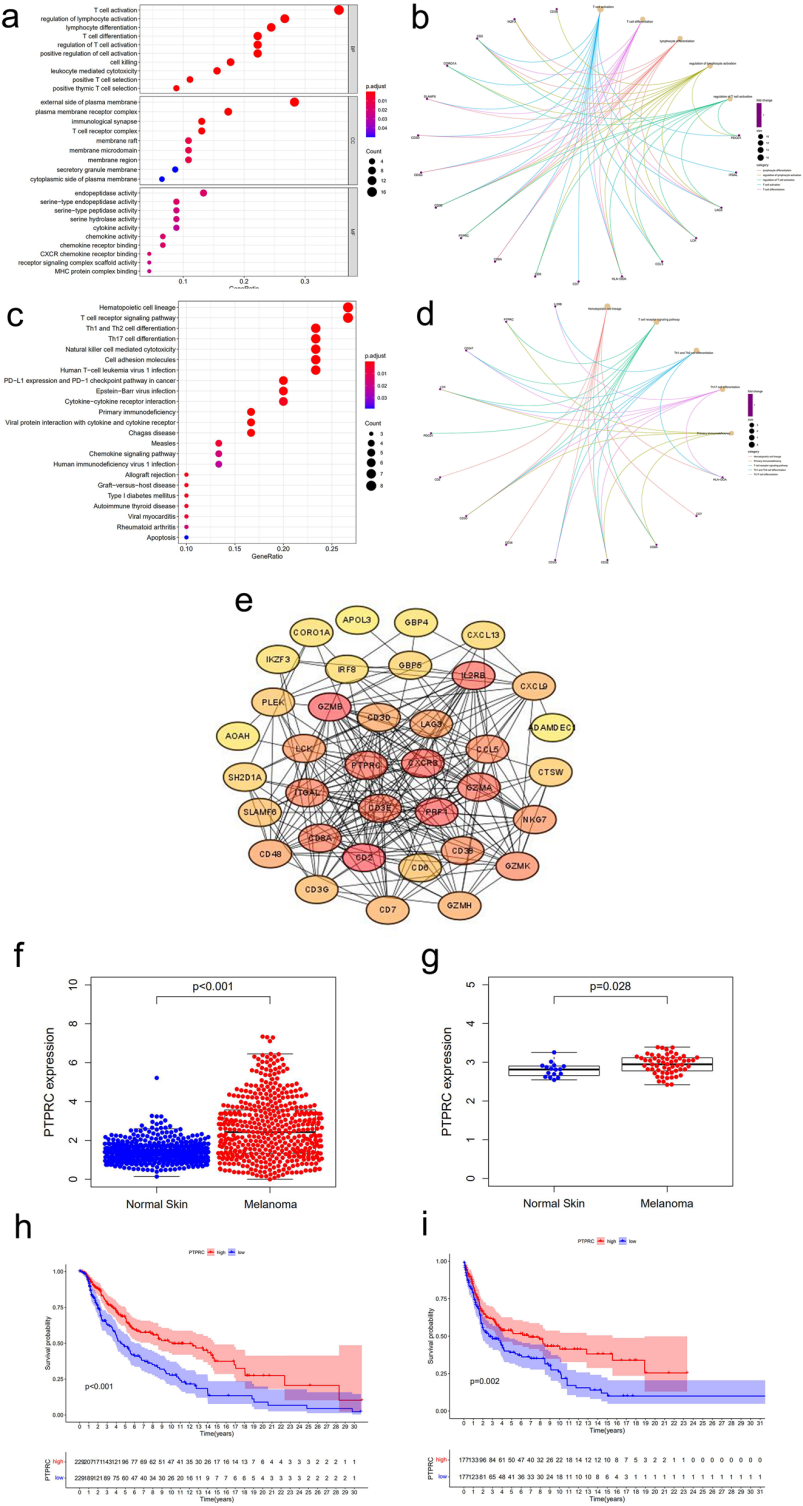
Enrichment analysis and PPI network of immune-related DEGs and the relationship between PTPRC expression and the prognosis of melanoma. (**a**) The top ten of GO-BP, GO-CC, and GO-MF. (**b**) The circos diagram of relationship between the DEGs and the main enrichment functions in GO. (**c**) 23 paths of the KEGG enrichment results. (**d**) The circos diagram of relationship between the DEGs and the main enrichment pathways in KEGG. (**e**) The PPI network based on the DEGs with |FC|> 2 and *P* < 0.05. (**f**,**g**) The differential expression of PTPRC between tumor and normal tissues in TCGA merged with GTEx and GEO. (**h**,**i**) Survival analysis of PTPRC in melanoma patients in TCGA and GEO.

GO analysis revealed that the 47 genes were primarily related to lymphocyte function, The results showed the top 10 biological processes (BP) GO terms, cellular component (CC) GO terms, molecular function (MF) GO terms (Fig. 3a), and the top 23 KEGG pathway terms (Fig. 3c). The correlation between the DEGs and the top 5 biological processes, including lymphocyte differentiation, regulation of lymphocyte activation, regulation of T cell activation, T cell activation and T cell differentiation is demonstrated (Fig. 3b). Similarly, KEGG enrichment analysis demonstrated a high enrichment of T cell function, such as Th1 and Th2 cell differentiation, Th17 cell differentiation, and T cell receptor signaling pathway (Fig. 3c,d). PPI network from the 47 genes was built using STRING network analysis, and then the core genes were examined employing the CytoHubba in Cytoscape software. Finally, the study identified PTPRC, one of the hub genes closely related to immune function, for further analysis (Fig. 3e). Furthermore, we established a co-expression network between the remaining 46 genes and PTPRC, and found that almost all of them were co-expressed with PTPRC (|correlation coefficient|> 0.6, *P* < 0.001), emphasizing the critical role of PTPRC in prognosis (Supplementary material Table S3).

Next, RNA-Seq data from 556 normal skin samples from the GTEx database and 470 melanoma samples from the TCGA were combined, and normal as well as disease cases from the GEO database were also analyzed, we observed that PTPRC was significantly overexpressed in melanoma (Fig. 3f,g), and higher levels of PTPRC expression in melanoma patients tended to have a better prognosis (Fig. 3h,i). In TCGA, the expression of PTPRC in patients under 65 years was higher than that in patients over 65 years old (Supplementary material Fig S2a, *P* = 0.01). Consistent with the trend of ImmuneScore, the expression of PTPRC significantly declined in T4 (Supplementary material Fig S2b). In contrast, the expression level decreased at stage II and then increased at stage III as the tumor stage increased (Supplementary material Fig S2c). In GEO, the expression of PTPRC did not change with age, and there was no significant difference in PTPRC expression between stage II and stage III (Supplementary material Fig S2d,e). The tumor mutation burden (TMB) is related to the prognosis and response to immunotherapy in specific cancer types. Here, we found that the high-TMB group had a significantly better prognosis (Supplementary material Fig S2f, *P* < 0.001). Of the 466 melanoma patients in TCGA, 46 (10%) carried somatic mutations in PTPRC, and patients with PTPRC mutations had a significantly higher TMB than wild-type samples (Supplementary material Fig S2i, *P* < 0.001), and the high-PTPRC group had a higher TMB (Supplementary material Fig S2h, *P* = 0.029). Kaplan–Meier analysis showed that PTPRC mutation was associated with a positive prognosis (Supplementary material Fig S2g, *P* = 0.008).

### The prognostic model efficiently predicts prognostic risk in melanoma patients

**Figure 4:**
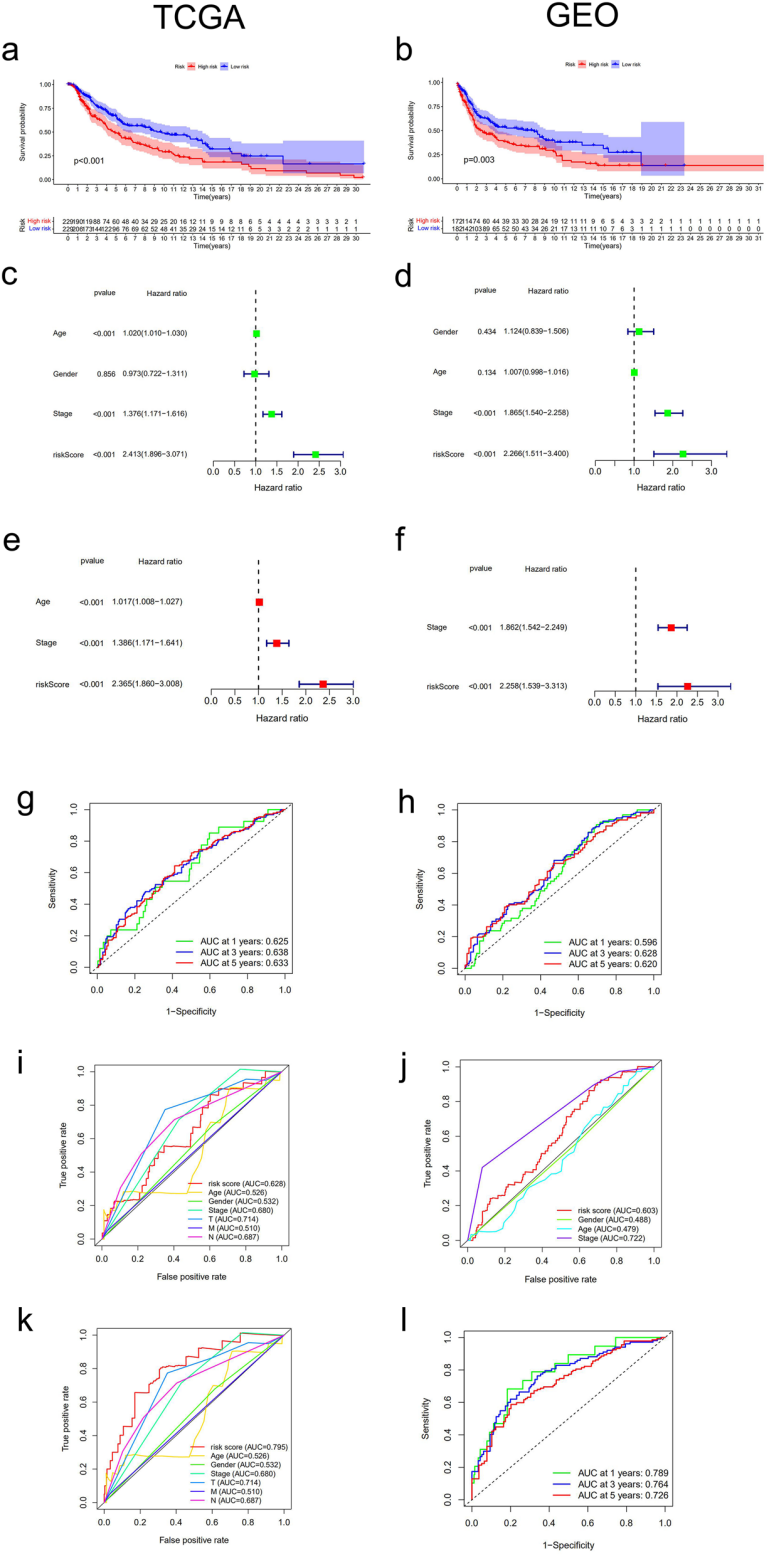
Survival analysis and Univariate and multivariate Cox regression of prognostic model in melanoma patients. (**a**,**b**) Kaplan–Meier survival curve of PTPRC expression in the training cohort and testing cohort. (**c**,**e**) Univariate and multivariate Cox regression of age, gender, stage and RiskScore (based on PTPRC expression) in training cohort. (**d**,**f**) Univariate and multivariate Cox regression of age, gender, stage and RiskScore (based on PTPRC expression) in testing cohort. (**g**,**i**) ROC curve analysis of RiskScore (based on PTPRC expression), age, gender and TNM classification in the training cohort. (**h**,**j**) ROC curve analysis of RiskScore (based on PTPRC expression), age, gender and TNM classification in the testing cohort. (**k**) ROC curve analysis of RiskScore (based on PTPRC expression combined with TNM classification), age, gender and TNM classification in the training cohort. (**l**)Time-dependent ROC in the testing cohort.

As previously mentioned, PTPRC overexpression associated with better prognosis of melanoma patients. A prognostic model based on the expression of PTPRC was constructed. The RiskScore of each patient was calculated, based on the median RiskScore, we categorized the samples into low- and high-risk clusters. Survival analysis showed that the high-risk cluster exhibited a significantly poorer OS rate than the low-risk cluster in both the training and testing groups (Fig. 4a,b). The RiskScore distribution, survival status, and survival time between two clusters were analyzed (Supplementary material Fig S3a–f). Univariate and multivariate Cox regression analyses indicated that, compared to other clinical characteristics such as age, gender, and stage, the RiskScore based on PTPRC expression could be an independent risk factor for OS in melanoma patients (Fig. 4c–f). However, AUC of the receiver operating characteristic curve (ROC curve) for the PTPRC signature did not demonstrate better efficiency in predicting melanoma patient survival risk than TNM classification (Fig. 4g–j). The study established ROC curves for other prognostic-related genes, but none of them showed higher prognostic accuracy. Our study then combined TNM classification and PTPRC expression to establish a prognosis model and found that the AUCs of the model significantly improved (Fig. 4k,l).

### PTPRC is closely linked to immune status of melanoma patients

**Figure 5:**
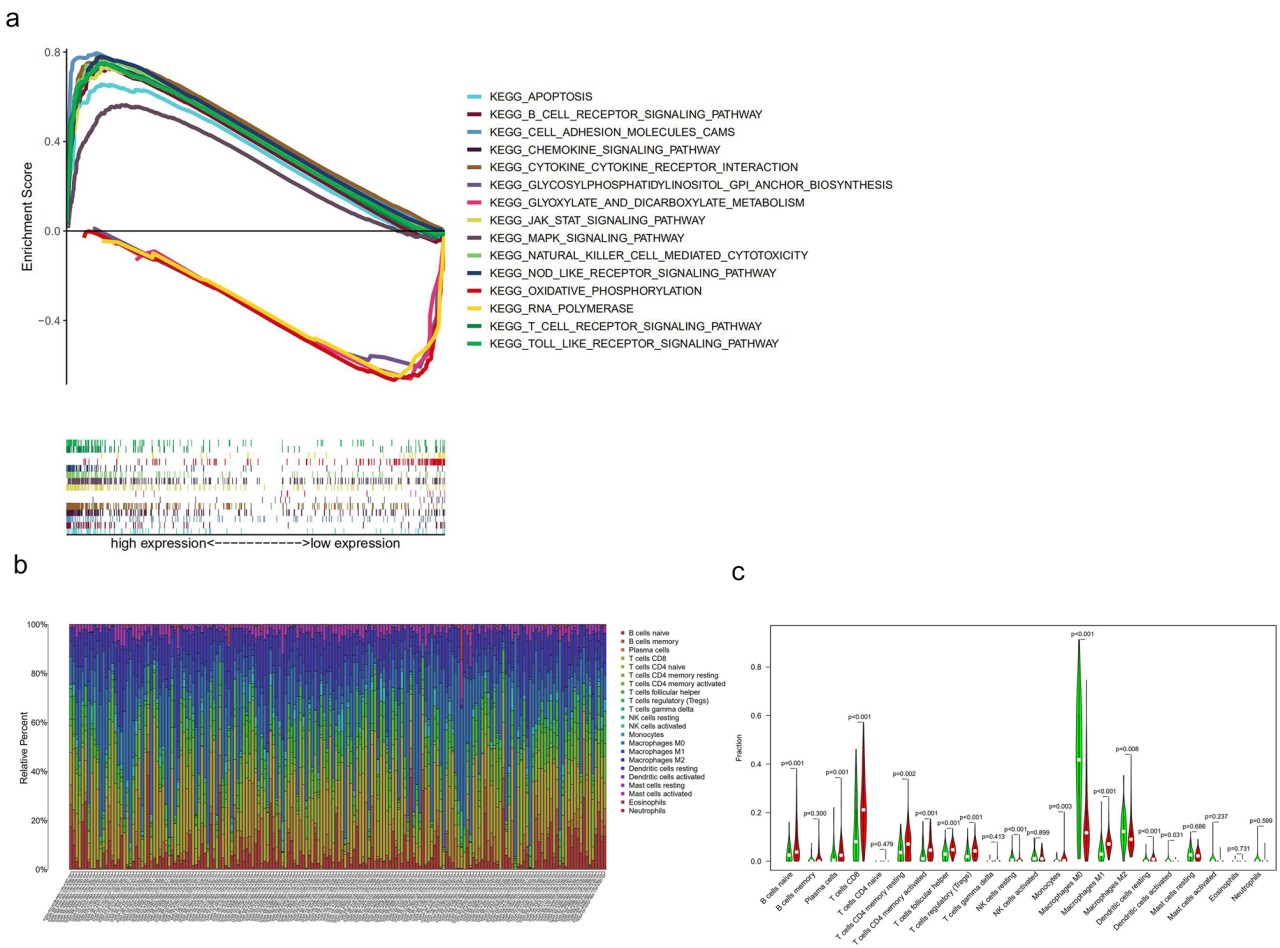
Relationship between PTPRC expression and immune infiltration. (**a**) The differences in enrichment pathways between high- and low-PTPRC groups. (**b**) Stacked bar chart of 22 immune cell types in each melanoma sample of TCGA. (**c**) Violin plot of the difference in immune cell infiltration between the high-PTPRC group and the low-PTPRC group. Green represents the low-PTPRC group, and red represents the high-PTPRC group.

As previously mentioned, PTPRC is an immune-related gene and is associated with prognosis of melanoma patients. GSEA analysis was conducted to further investigate the differences in enrichment pathways between the high- and low-PTPRC groups. We discovered that pathways enriched in the high-PTPRC group were mainly related to immune-related pathways, including B cell receptor signaling pathways, cell adhesion molecules, cytokine signaling pathways, cytokine-cytokine receptor interactions, natural killer cell-mediated cytotoxicity, and T cell receptor signaling pathways. In contrast, pathways enriched the low-PTPRC group was associated with metabolisms, such as glycosylphosphatidylinositol anchor biosynthesis, RNA polymerase, oxidative phosphorylation, glyoxylate, and dicarboxylate metabolism (Fig. 5a). To explore the association between PTPRC and tumor-infiltrating immune cells in the TME of melanoma, the infiltration of 22 types of immune cells were analyzed in the TCGA database (Fig. 5b). 14 of the 22 immune cell types was significantly different between the high- and low- PTPRC groups (Fig. 5c). The expression of PTPRC was positively correlated with activated memory B cells, naive plasma cells, CD8 T cells, CD4 memory resting T cells, CD4 memory activated T cells, T follicular helper cells, T regulatory cells (Tregs), monocytes, M1 macrophages, and resting dendritic cells and negatively correlated with M0 macrophages, M2 macrophages, resting natural killer cells, and activated dendritic cells (Supplementary material Fig S4a).Furthermore, the correlation matrix showed that neutrophils had the strongest positive correlation with activated mast cells, while M0 macrophages had a negative correlation with CD8 T cells (Supplementary material Fig S4b).

### PTPRC-associated risk model predicts immunotherapy efficacy in melanoma

**Figure 6:**
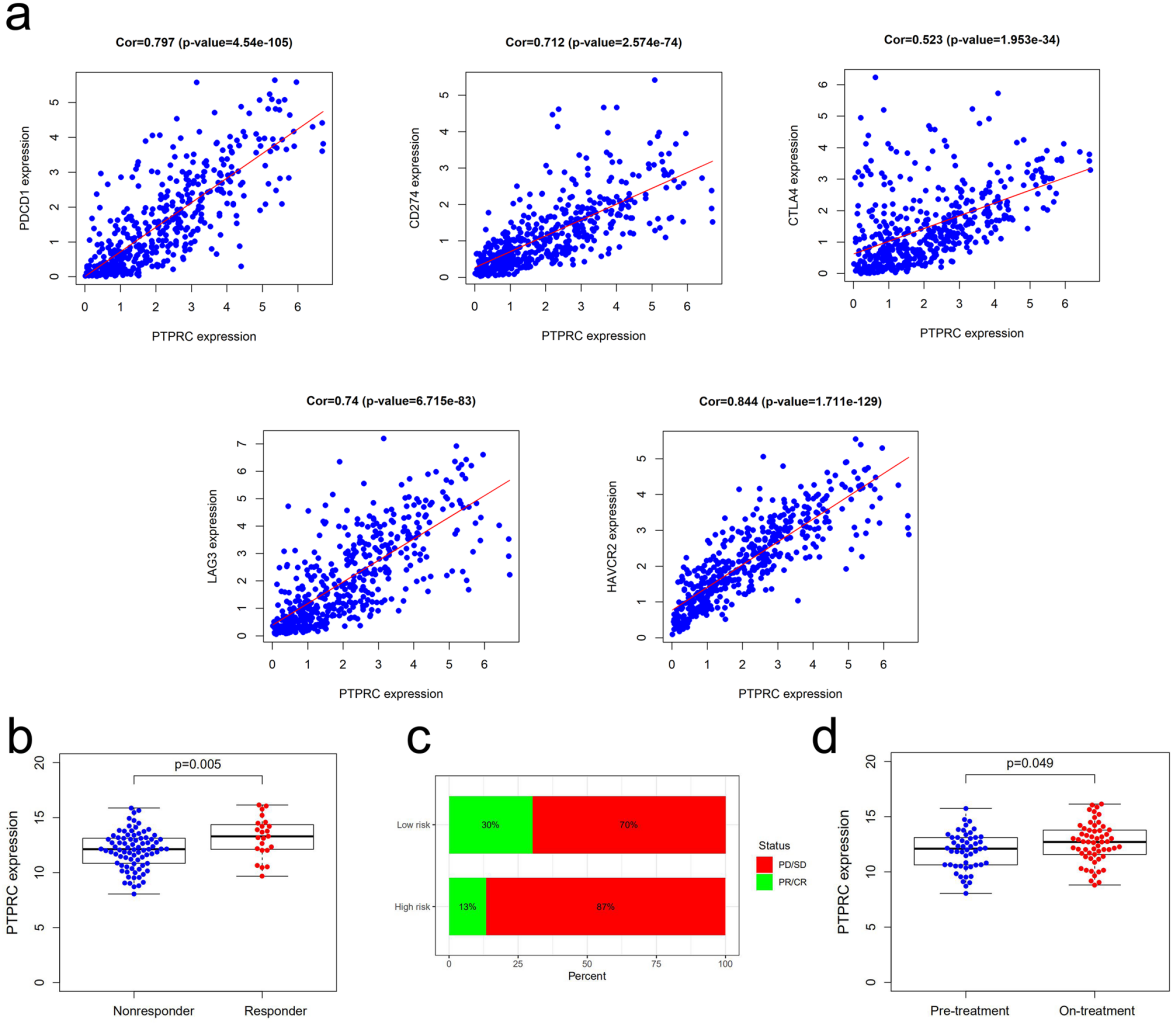
The relationship between PTPRC and immunotherapy. (**a**) Co-expression relationship between PTPRC and several ICPs. (**b**) PTPRC expression in responders and non-responders. (**c**) The proportion of responders (Partial response, PR/Complete response, CR) in high and low-risk groups. (**d**) The expression of PTPRC before and after immunotherapy.

To assess the potential of PTPRC as a predictor of immunotherapy response, our study investigated the relationship between PTPRC expression and common immune checkpoint molecules, including PDCD1, PD-L1/CD274, CTLA4, LAG3, and HAVCR2. Our findings indicated a positive correlation between PTPRC expression and the levels of these immune checkpoint molecules (Fig. 6a). We analyzed clinical data from the GEO database (GSE91061) and observed that the expression of PTPRC was significantly higher in responders compared to non-responders (Fig. 6b). Moreover, patients with high PTPRC expression had a higher probability of responding to immune checkpoint therapy, with the percentage of responders being 30% in the low-risk group and 13% in the high-risk group (Fig. 6c). Lastly, we evaluated the impact of anti-CTLA4 and anti-PD1 therapy on PTPRC expression and found that the expression levels of PTPRC increased significantly post-therapy (*P* = 0.049) (Fig. 6d).

### Low PTPRC expression promotes migration, invasion and proliferation of melanoma cell lines

**Figure 7:**
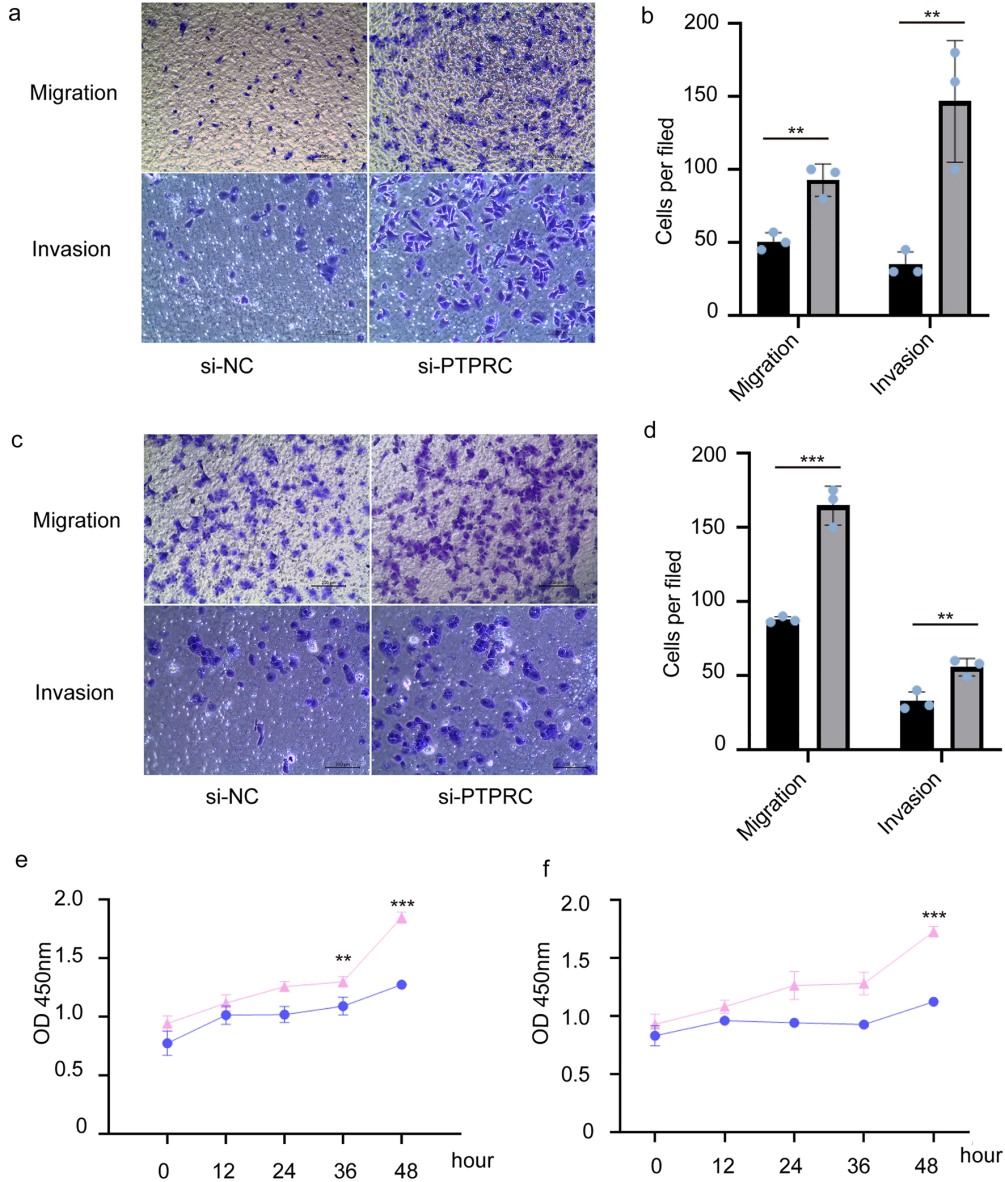
Effect of knocking down PTPRC expression on the migration, invasion, and proliferation ability of A375 and MEL-28 cells. Transwell assays carried out in A375 (**A**, si-NC, black; si-PTPRC, grey) and MEL-28(**B**, si-NC, black; si-PTPRC, grey) cell lines. Cell proliferation assay of A375 (**C**, si-NC, blue; si-PTPRC, pink) and MEL-28(**D**, si-NC, blue; si-PTPRC, pink) cell lines. *P < 0.05, ***P < 0.01, ***P < 0.001.

Next, to determine whether PTPRC affects migration, invasion and proliferation of melanoma cells, siRNA targeted PTPRC was applied to knockdown PTPRC expression in A375 and MEL-28 cell lines (Supplementary material Fig S5a,b, original blots are presented in Supplementary FigS6a,b). The results show that silencing of PTPRC significantly promoted migration, invasion, and proliferative cell number of cancer cells in A375 and MEL-28 cell lines compared to controls (Fig. 7).

### Verification of PTPRC expression in melanoma tissues

**Figure 8:**
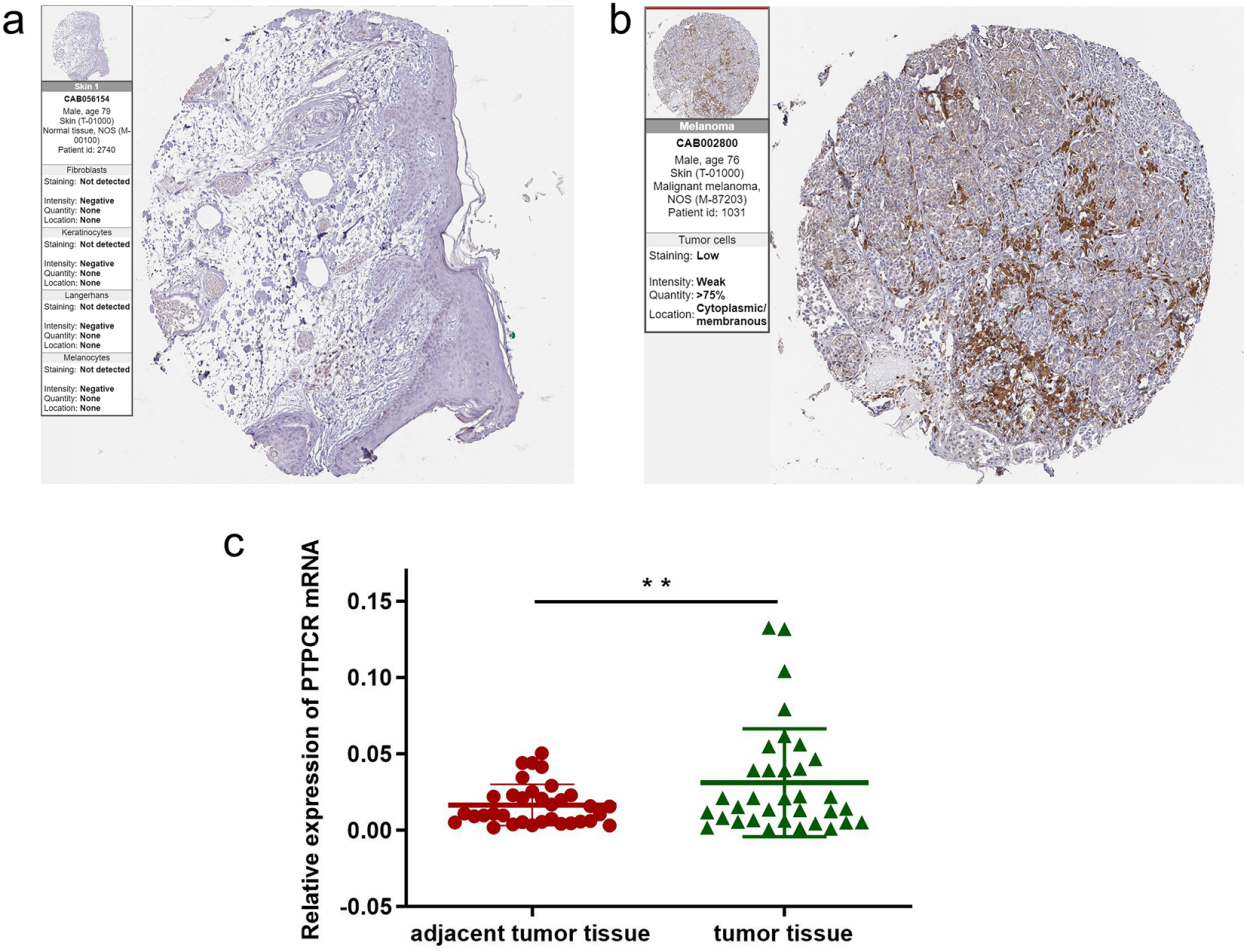
The expression of PTPRC in melanoma patients. (**a**) The protein expression of PTPRC according to HPA. (**b**) The mRNA expression level of PTPRC in 34 pairs of melanoma tumor and adjacent normal tissues was measured by qPCR. *P < 0.05, ***P < 0.01, ***P < 0.001.

According to immunohistochemical data from HPA, the protein expression of PTPRC is higher in melanoma tissues than that in normal (Fig. 8a,b). And the mRNA expression of PTPRC was upregulated in melanoma compared to adjacent tumor tissue as verified by Real-time quantitative PCR (qPCR) (Fig. 8c).

## Discussion

Melanoma is an aggressive skin cancer with increasing morbidity and mortality. Although current immunotherapy has made significant progress, there are still many melanoma patients who do not respond to immunotherapy or who experience adverse reactions^17^. As a result, new biomarkers for melanoma patients are required to provide a more precise prediction of patient survival and response to immunotherapy. Also, given that TME influences and determines tumor growth, treatment responsiveness, and patient prognosis^18,19^, this study explores an immune-related gene (PTPRC) in TME that has good predictive power for both prognosis and response to immunotherapy in melanoma patients through a comprehensive bioinformatics analysis.

PTPRC, also known as CD45, is expressed in almost all hemopoietic cells except mature red blood cells and is an essential regulator of T and B cell antigen receptor-mediated activation. It is also an important protein on the cell surface of the blood and immune system^20^. It controls immune function by regulating lymphocyte survival, cytokine responses, and T cell activation signaling. Altering PTPRC can lead to severe combined immunodeficiency^21^. In addition, CD45 activity is essential for an efficient immune response. In particular, in NK cells, CD45 expression is highly correlated with NK cell maturation^22,23^. As a gene closely related to immune function, PTPRC has consistently been reported to be related to autoimmune diseases such as systemic lupus erythematosus and multiple sclerosis^24^. It was also reported that PTPRC was associated with favorable disease-specific survival in lung cancer^25–27^. However, the function and mechanism of PTPRC in melanoma have not been elucidated.

In line with other reports, we found that the immune component of TME plays a critical role in the clinical outcome of melanoma patients, including survival and TNM stage classification. Then, a comprehensive and systematic analysis of the data using various databases showed that high expression of PTPRC was significantly associated with a better prognosis, while a RiskScore calculated from PTPRC expression revealed a worse prognosis in the high-risk group, suggesting that PTPRC expression was expressed by infiltrating immune cells rather than cancer cells present in the tissue, which is associated with an immune response. To demonstrate the possible protective role of PTPRC in melanoma development, we showed that low expression of PTPRC enhanced the migration, invasion, and proliferation of tumor cells by knocking down the expression level of PTPRC in the melanoma cell lines A375 and MEL-28. Next, consideration of PTPRC expression based on TNM classification effectively improved the reliability of predicting the prognosis of melanoma patients, and thus the model may be able to provide a prediction of the prognosis of melanoma patients in the clinic.

Tumor-infiltrating lymphocytes determine tumor progression and aggressiveness and are a source of important prognostic information for patients. Analysis by GSEA and CIBERSORT showed that the PTPRC high expression group was significantly associated with immune-related biological processes and activated immune infiltrating cells, which is consistent with previous studies. Furthermore, PTPRC was co-expressed with a variety of immune checkpoint genes, including PDCD1, PD-L1/CD274, CTLA4, LAG3, and HAVCR2. PTPRC expression levels increased significantly after anti-CTLA4 and anti PTPRC expression levels increased significantly after anti-CTLA4 and anti-PD1 treatment, probably because inhibition of these checkpoints resulted in activation of the PTPRC-expressing immune cell population. Also, PTPRC expression is positively correlated with TMB, and studies have shown that tumors with high TMB are thought to be more susceptible to immune checkpoint inhibitors (ICI) as they express more neoantigens and are therefore more likely to be recognized and targeted by T cells^28^. These results all suggest that PTPRC may play an important role in predicting and improving the response of melanoma to immunotherapy. Finally, we confirmed by immunohistochemistry results in HPA and qPCR that PTPRC expression is higher in melanoma than in normal skin.

In recent years, significant developments in molecular analysis, genomics, and cancer biology have led to the discovery of many new cancer biomarkers. Cancer biomarkers can be classified into three categories: diagnostic, prognostic, or predictive. Lactate dehydrogenase (LDH) and S100B have been found in research to be the most useful serum prognostic indicators. In metastatic melanoma, serum LDH is a potent independent prognostic indicator, and increased LDH is linked to a lower OS in patients with advanced disease. To date, LDH is the only widely used prognostic serum marker for melanoma^29,30^. Recently, high LDH has been linked to a poor response to anti-PD1 therapy, implying that it could be used as a predictive biomarker^31^. However, this remains to be proven. Numerous studies have shown that elevated serum S100B levels are associated with increased aggressiveness and decreased survival in melanoma, suggesting that S100B can be used as a prognostic marker^32–35^, S100B is also valuable for monitoring patients during treatment, with increased levels associated with disease progression and decreased levels associated with disease regression^34,36^. However, given the low incidence of elevated serum levels of S100B in patients with early-stage disease, S100B was not considered useful for screening or early detection. Nevertheless, the two predominant serum markers are elevated in most non-neoplastic diseases and are susceptible to a variety of confounding factors when making judgments. With the boom in immunotherapy and its revolutionary improvements in the treatment of melanoma, there is an urgent need for appropriate prognostic biomarkers to estimate risk and an even more urgent need for predictive biomarkers to determine which patients are likely to respond to immunotherapy. Studies have shown that TMB^37,38^, IFN γ-related gene expression profile^39,40^, infiltration of CD8 + TILs^41,42^ T, and HLA heterozygosity^43,44^ all have a possible role in predicting response to ICI, but none have been found to correlate with prognosis. As for PTPRC, which was studied in this paper, it also predicts prognosis and responsiveness to immunotherapy. In conclusion, it makes sense that PTPRC has been proposed as a biomarker for melanoma.

Overall, we identified the value of PTPRC as an indicator of TME status remodeling in melanoma and also as a potential predictor of response to immunotherapy. In addition, the model created in combination with TNM classification allows for more precise stratification of patient survival. It is important to note that while these results suggest a clear clinical link between PTPRC expression and melanoma, it has the limitation that only data from various databases the validation of mRNA levels and some simple in vitro experiments. Therefore, further additional in vivo and in vitro studies are necessary to seek results that logically support these clinical associations.

## Conclusion

This study identifies a new immune-related prognostic indicator for use in melanoma that may also serve as a potential predictor of response to immunotherapy. Targeting PTPRC may be a treatment approach for melanoma based on the hallmark immune infiltration landscape observed in our study.

## Materials and methods

### Raw data acquisition

RNA-Seq data and clinical information of 470 melanoma patients were obtained from The Cancer Genome Atlas (TCGA) (https://portal.gdc.cancer.gov/) as a training set. The standard skin samples were retrieved from Genotype-Tissue Expression Project (GTEx) (https://www.genome.gov/Funded-Programs-Projects/Genotype-Tissue-Expression-Project). Another 332 melanoma patients’ information obtained Gene Expression Omnibus (GEO) datasets (https://www.ncbi.nlm.nih.gov/geo/) (data merged from GSE15605, GSE19234, GSE22154, GSE54467 and GSE65904) was used as a testing set. Somatic mutation information for melanoma patients was obtained from TCGA and International Cancer Genome Consortium (ICGC) (https://dcc.icgc.org/). The results of melanoma single-cell sequencing were also obtained from GEO datasets. Batch corrections were applied when different cohorts were combined. All of the above datasets are accessible to the public.

### Evaluation of ImmuneScore, StromalScore, and ESTIMATEScore

The immune infiltration (ImmuneScore), overall stromal content (StromalScore), and combined (ESTIMATEScore) of each melanoma sample were calculated using ESTIMATE algorithms^45^.

### Identification of differentially expressed genes (DEGs) in high- and low-immunity groups

Samples were divided into high- and low- immune score groups, and DEGs were identified based on the conditions: |log2 fold change (log2 FC) |> 1.0, false discovery rate (FDR) < 0.05 using the “limma” R package. The “pheatmap” R package was used to generate heatmaps.

### Analysis of co-expression module construction in melanoma

The power value was filtered out in the module construction through weighted gene co-expression network analysis (WGCNA). The independence and average connectivity of different modules were tested using the gradient method (power values ranging from 1 to 20). When the degree of independence arrived at 0.8, the appropriate power value was determined, and then the module construction was carried out through the WGCNA. In addition, the corresponding genetic information for each module was extracted. The minimum number of genes was set to 50. A Venn diagram was used to intersect the key gene module closely associated with the high-immunity in melanoma and the genes differentially expressed between the high-immunity and low-immunity groups.

### Survival analysis

Survival analysis was performed through the “survminer” and “survival” R packages. Kaplan–Meier (K–M) survival curves were drawn to analyze the relationship between DEG expression and the overall survival (OS) of melanoma patients.

### Functional enrichment analysis

The enrichment analysis of Gene Ontology (GO) and Kyoto Encyclopedia of Genes and Genomes (KEGG) were carried out by “clusterProfiler,” “enrichplot,” and “ggplot2” packages in R. GO results included molecular function (MF), biological process (BP), and cellular component (CC). FDR < 0.05 was statistically significant.

### Protein–protein interaction (PPI) network analysis

Through PPI network analysis of the STRING website (https://cn.string-db.org/), the interaction between cross genes was obtained. Core genes were identified using the CytoHubba plug-in of Cytoscape (http://www.cytoscape.org) (version 3.9.1) with the highest confidence (0.70) as a threshold.

### Correlation analysis of scores with clinicopathological characteristics

Software package “ggpubr” in R was used to analyze the correlation between the scores and clinicopathological features. The statistical significance was determined using either the Wilcoxon rank-sum test or the Kruskal–Wallis rank-sum test.

### Somatic mutation analysis

To assess the tumor mutation burden (TMB) between the PTPRC wild-type and mutant groups, the nonparametric Wilcoxon rank-sum test was used. A two-sided *P* < 0.05 was considered statistically significant. Kaplan–Meier curves and the log-rank test were utilized to assess the survival differences between patient groups with different mutation statuses and TMB.

### Establishment of a risk model to evaluate the riskScore

Univariate and multivariate Cox regression analyses were performed to confirm whether this gene could serve as an independent clinical prognostic predictor. Then the Risk Model was constructed. The area under curve (AUC) values were calculated for each model, and the corresponding 1 -, 3 -, and 5-year ROC curves were plotted. The following formula was used to calculate the riskScore from the risk model for all clinical cases: RiskScore = ∑ki = 1βiSi.

### Gene set enrichment analysis (GSEA) and Immune cell infiltration analysis

GSEA was performed to explore the potential mechanisms of genes affecting prognosis, and obtain the signaling pathways of up-regulation and down-regulation. FDR < 0.05 was considered statistically significant. The infiltration of 22 immune cell types in tumor tissues between the low- and high-risk groups were estimated by CIBERSORT.

### Analysis of immunotherapeutic benefits and PTPRC

To study the relationship between the PTPRC and the expression level of immunosuppressive molecules related to ICIs, we used “limma” package to perform the co-expression correlation analysis of PTPRC with ICIs. Gene expression data with immunotherapy was downloaded from the GEO database (GSE91061) and analyzed to determine the expression level between responders and non-responders. The R package Seurat was used for most preprocessing steps for the scRNA-seq datasets downloaded from the GEO database (GSE120575). The batch effect was removed at first, and then t-SNE for data visualization was calculated with fast interpolation-based t-SNE.

### Human protein atlas (HPA)

The immunohistochemistry expression graph of related genes was obtained from the HPA database (https://www.proteinatlas.org/).

### Cell culture

The human tumor cell line A375 (CL-0014) and MEL-28 (CL-0717) were purchased from Procell. Cells were cultured in DMEM medium (C3113-0500, Vivacell Biosciences) supplemented with 10% FBS (Excell, FSP500) and 1% 10 kU/ml penicillin/10 mg/ml streptomycin (Procell, PB180120) at 37 °C with 5% CO_2_ in a humidified incubator. Cells from generations 3–4 were utilized for subsequent experiments.

### Transfection of malignant melanoma cell

The transfection reagent mixture was prepared as follows: 5 μL of LipofectamineTM2000 (ThermoFisher Scientific, America), 250 μL of serum-free medium, 5 μL of negative control siRNA or PTPRC siRNA (2 μg), and 250 μL of serum-free medium. A375 and MEL-28 cells transfected with PTPRC siRNA (5′-GCTGCACATCAAGGAGTAATT-3′) were named si-PTPRC group, and transfected with negative control (NC) siRNA were named si-NC group. The reagents were mixed well in two EP tubes, and left for 20 min. The complete culture medium in the corresponding wells was discarded, and the mixed transfection reagents were added, and the corresponding treatment was carried out 48 h later. The transfection efficiency was determined by the expression levels of protein (13917 T, purchased from Cell Signaling Technology) and mRNA.

### Cell counting Kit-8 (CCK8)

After transfection with si-NC or si-PTPRC in A375 cells and MEL-28 cells, the cells were seeded into 96-well plates (5 × 10^3^ cells/well). Thereafter, the cell viability was assessed using CCK-8 (China Elabscience Biotechnology) at 0, 12, 24, 36, and 48 h according to the manufacturer’s instructions.

### Migration assay

After transfection of A375 cells and MEL-28 cells with si-NC or si-PTPRC, cell migration assays were performed using 24-well Transwell plates (8.0 mm; Corning, NY, USA). Cancer cells (5 × 10^4^, A375-si-NC, A375-si-PTPRC, MEL-28-si-NC, MEL-28-si-PTPRC) were implanted into the upper chamber, and 600 μL of DMEM containing 10% FBS was placed into the lower chamber. The Transwell plates were then incubated at 37 °C in a 5% CO_2_ incubator for 48 h, fixed with 4% formaldehyde for half an hour, and stained with 0.01% crystal violet. Unmigrated cells were carefully removed with a cotton swab, and then the cells that migrated into the lower chamber were counted under a microscope.

### Patients and specimens

This study was approved by the Ethics Committee of the Third Xiangya Hospital of Central South University, and all melanoma patients participating in the study signed an informed consent form. For expression analysis, a total of 34 pairs of melanoma tissues and corresponding peritumoral normal tissues were immediately frozen in liquid nitrogen until RNA extraction.

### RNA extraction and real-time quantitative PCR (qPCR)

Total RNA was obtained from melanoma and adjacent tumor tissues using TRIzol reagent (Invitrogen). Reverse transcriptase reactions were performed using the PrimeScript™ RT kit (Takara). β-Actin was used to normalize the expression levels of mRNAs for genes. Normalized CT values were used to calculate ploidy differences between groups. Primer sequences are shown in Supplementary material Table S1.

### Statistical analysis

All bioinformatic statistical analyses were performed using the R software package version 4.2.3. Kaplan–Meier survival analysis and the log-rank test were used to derive prognostic values and evaluate patient survival in different subgroups in each dataset. Or continuous data, the non-parametric Wilcoxon rank sum test was employed to examine the connection between the two groups. The Kruskal–Wallis test was used to compare more than two groups. Using univariate and multivariate Cox regression (R package “survival”), clinical characteristics from the high and low-risk groups were evaluated for prognostic factors. Spearman correlation analysis was used to assess correlation coefficients. Experimental data were analyzed using GraphPad Prism 9, and *P* < 0.05 was considered statistically significant.

### Ethical approval

This study was performed in line with the principles of the Declaration of Helsinki. Approval was granted by the Ethics Committee of The Third Xiangya Hospital of Central South University. And informed consent has been obtained from all subjects and/or their legal guardians.

### Supplementary Information

Supplementary Information 1.

## Data Availability

No sequencing was performed in this study, the datasets generated during and/or analysed during the current study are available in the TCGA database (https://www.cancer.gov/ccg/research/genome-sequencing/tcga), GEO database (https://www.ncbi.nlm.nih.gov/geo/) (GSE15605, GSE19234, GSE22154, GSE54467, GSE65904 and GSE91061) and Genotype-Tissue Expression Project (GTEx) (https://www.genome.gov/Funded-Programs-Projects/Genotype-Tissue-Expression-Project).
