# Supplementary material for: PTPRC functions as a prognosis biomarker in the tumor microenvironment of cutaneous melanoma
Source: Sci Rep. 2023 Nov 23;13:20617. doi: 10.1038/s41598-023-46794-6 (PMC10667527; doi:10.1038/s41598-023-46794-6)
Supplement: Supplementary file 1 — Supplementary Information 1. [file 41598_2023_46794_MOESM1_ESM.pdf]

Supplementary materials

PTPRC Functions as a Prognosis Biomarker in the Tumor Microenvironment of cutaneous melanoma

Xuemei Li[1], Zhanghui Yue[1], Dan Wang[1*], Lu Zhou[1*]

[1] Department of Dermatology, The Third Xiangya Hospital, Central South University, Changsha, Hunan Province, 410000 P. R. China

#These authors contributed equally to this work.

*** Correspondence:**
Corresponding Author: Dr. Dan Wang, email: 168301013@csu.edu.cnz; Lu Zhou, email: 290991732@qq.com

Supplementary material

Table S1 primers used for RT-PCR

Transcript	Forward primer (5”-3)	Reverse primer (5”-3)
PTPRC	AGCCAATCCAAGTCAC CAA	AGCCAATCCAAGTCAC CAA

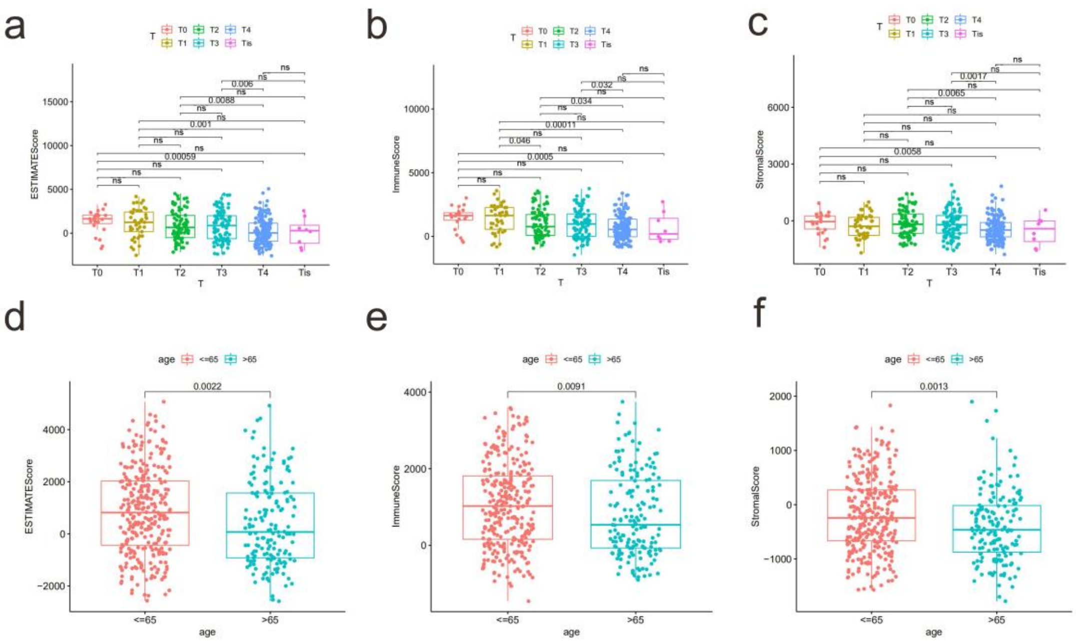

Fig S1. Relationship between T-staging and age and the three scores.

(a-c) The boxplot of ESTIMATEScore, ImmuneScore, and StromalScore of cutaneous melanoma patients in different T Staging in TCGA.

(d-f) The boxplot of ESTIMATEScore, ImmuneScore, and StromalScore of cutaneous melanoma patients in different age groups in TCGA.

Table S2 Univariate Cox proportional hazards regression analysis

Gene	HR	HR.95L	HR.95H	p-value
CD69	0.920705151	0.862685173	0.982627267	0.012857869
PDCD1	0.941161368	0.9113183	0.971981711	0.000225545
GBP5	0.954620399	0.933479016	0.976240591	0.0000482
NKG7	0.993270924	0.989183455	0.997375284	0.001331387

ITGAL	0.968135281	0.950457251	0.986142112	0.000572929
GZMK	0.970980328	0.953039021	0.989259387	0.001969463
LAG3	0.969734241	0.954280004	0.985438754	0.000177162
IL2RB	0.968162596	0.950324538	0.986335483	0.000649496
LCK	0.965865818	0.945845984	0.986309392	0.001154351
CTSW	0.966525056	0.947568446	0.985860903	0.000754464
CXCL9	0.99581603	0.993685998	0.997950628	0.000124175
PRF1	0.982799325	0.972559288	0.993147179	0.001167376
CCL5	0.995615324	0.993296113	0.997939949	0.000221585
CD48	0.970723641	0.953532112	0.988225122	0.001117355
IRF8	0.964847893	0.940712892	0.989602104	0.005628791
GBP4	0.981103208	0.973270051	0.988999408	0.00000309
HLA-DOA	0.982708	0.973182559	0.992326675	0.000448182
NAPSB	0.981036792	0.964899938	0.997443516	0.023669122
CD7	0.96188935	0.942574848	0.98159963	0.000173707
CD247	0.9096602	0.86260011	0.959287705	0.000476628
CXCR3	0.95685575	0.932565629	0.981778545	0.000774667
GZMA	0.98172593	0.972405485	0.991135712	0.000151036
CD6	0.931441923	0.889190652	0.975700829	0.002712627
GNLY	0.95882332	0.926979876	0.991760643	0.014684207
ADAMDEC1	0.966219362	0.944719079	0.988208957	0.002762236
SIGLEC10	0.905520216	0.860555994	0.952833828	0.000133858
IKZF3	0.941250495	0.907084487	0.976703392	0.00132956
SLA	0.90546667	0.86359765	0.949365588	0.0000394
CD8A	0.977835239	0.965978943	0.989837058	0.00031683
TBC1D10C	0.931502094	0.892774702	0.971909429	0.001056386
PTPRC	0.977238591	0.963208651	0.99147289	0.001804493
CD3E	0.985725041	0.976745756	0.994786873	0.002074

CD3G	0.904917532	0.850552741	0.962757159	0.001574416
GZMB	0.978691115	0.966657068	0.990874976	0.000644534
SH2D1A	0.90549217	0.858509832	0.955045637	0.000260239
CXCL13	0.98778543	0.980702731	0.994919281	0.000816035
APOL3	0.946388086	0.92441046	0.968888224	0.0000043
CD38	0.89427349	0.849360687	0.941561209	0.0000213
PLEK	0.975012726	0.962987128	0.987188496	0.0000643
CD3D	0.983148538	0.973903934	0.992480895	0.000422281
GZMH	0.96935438	0.952392681	0.986618161	0.000548749
IL32	0.982167076	0.971782302	0.992662825	0.000907193
SLAMF6	0.944286477	0.91367781	0.97592055	0.000650253
CORO1A	0.989213339	0.982506203	0.995966262	0.001781779
AOAH	0.94043041	0.907357714	0.974708588	0.000772726
CD2	0.98237255	0.973315769	0.991513605	0.000167584
ARHGAP9	0.930058105	0.891707036	0.970058599	0.000738526

Table S3 Co-expression analysis of PTPRC

gene1	gene2	correlation coefficient	p-value
PTPRC	CD69	0.905	2.5143E-176
PTPRC	PDCD1	0.797	4.5396E-105
PTPRC	GBP5	0.88	1.1106E-153
PTPRC	NKG7	0.755	2.56444E-88
PTPRC	ITGAL	0.905	4.3085E-176
PTPRC	GZMK	0.888	4.7878E-161
PTPRC	LAG3	0.74	6.71541E-83
PTPRC	IL2RB	0.865	1.1761E-142
PTPRC	LCK	0.904	1.4791E-175

PTPRC	CTSW	0.75	2.32618E-86
PTPRC	CXCL9	0.834	2.9561E-123
PTPRC	PRF1	0.777	1.45018E-96
PTPRC	CCL5	0.769	3.06321E-93
PTPRC	CD48	0.907	2.1694E-178
PTPRC	IRF8	0.949	7.2241E-238
PTPRC	GBP4	0.78	6.39373E-98
PTPRC	HLA-DOA	0.84	7.8674E-127
PTPRC	CD7	0.795	6.9937E-104
PTPRC	CD247	0.896	1.1518E-167
PTPRC	CXCR3	0.798	2.1021E-105
PTPRC	GZMA	0.835	6.0477E-124
PTPRC	CD6	0.869	5.4604E-146
PTPRC	GNLY	0.609	3.25489E-49
PTPRC	ADAMDEC1	0.86	3.5511E-139
PTPRC	SIGLEC10	0.881	1.4535E-154
PTPRC	IKZF3	0.919	5.6179E-192
PTPRC	SLA	0.93	2.5708E-206
PTPRC	CD8A	0.834	3.1987E-123
PTPRC	TBC1D10C	0.864	8.1493E-142
PTPRC	CD3E	0.861	2.8364E-140
PTPRC	CD3G	0.939	1.4559E-220
PTPRC	GZMB	0.718	5.88607E-76
PTPRC	SH2D1A	0.935	6.6887E-214
PTPRC	CXCL13	0.861	3.3183E-140
PTPRC	APOL3	0.867	1.7252E-144
PTPRC	CD38	0.88	6.3388E-154
PTPRC	PLEK	0.938	3.8977E-218

PTPRC	CD3D	0.869	7.105E-146
PTPRC	GZMH	0.703	1.43253E-71
PTPRC	IL32	0.773	4.47096E-95
PTPRC	SLAMF6	0.925	4.1644E-200
PTPRC	CORO1A	0.863	1.5357E-141
PTPRC	AOAH	0.901	4.5979E-173
PTPRC	CD2	0.893	3.7789E-165
PTPRC	ARHGAP9	0.904	2.2886E-175

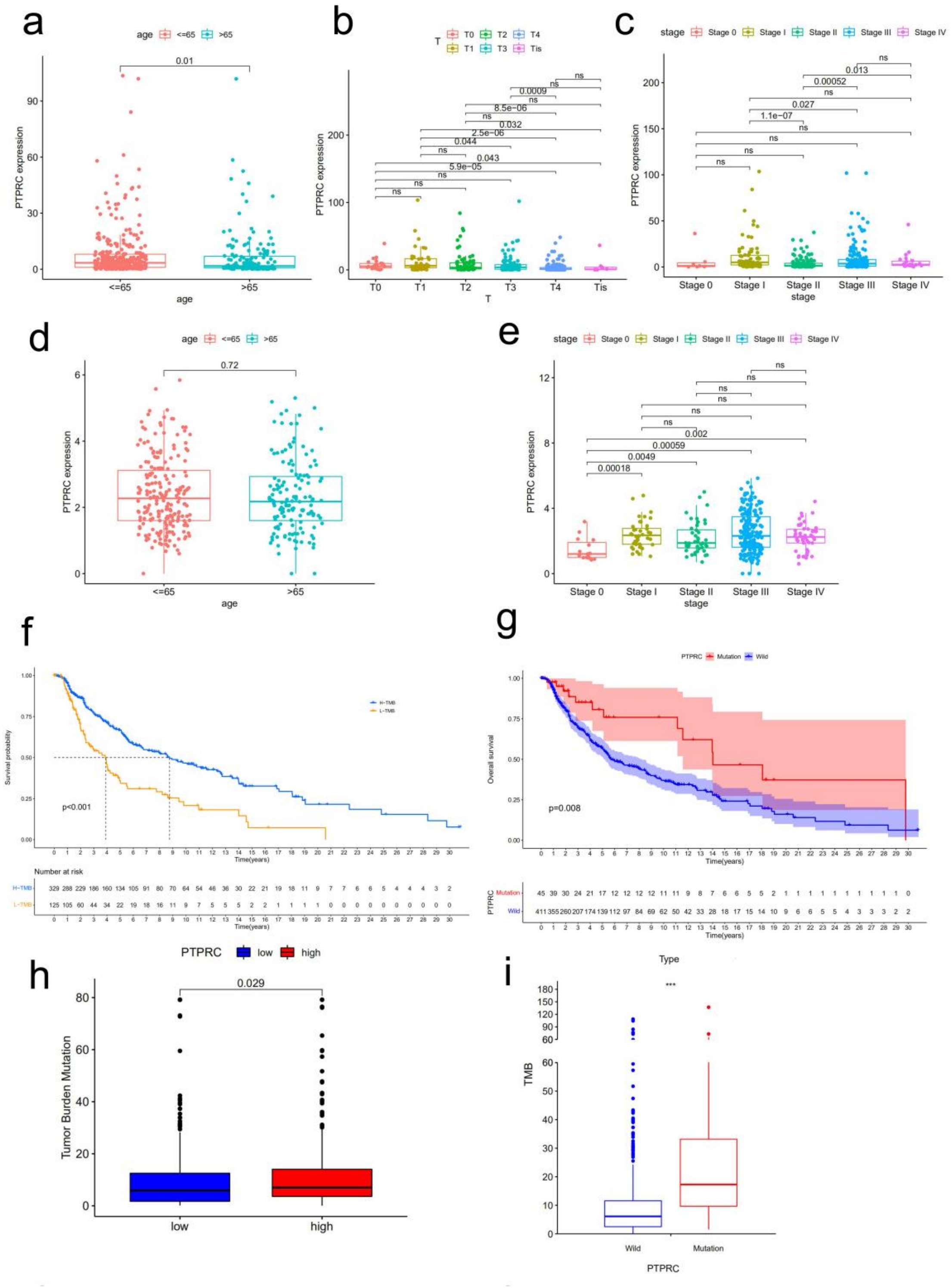

FigS2. Analysis of PTPRC in patients with melanoma.

(a, b, c) Distribution of the expression level of PTPRC of age, T and stage in TCGA. **(d, e)** Distribution of the expression level of PTPRC of age and stage in GEO. **(f)** The survival curve of the high-TMB group. **(g)** Survival analysis of PTPRC mutation in melanoma patients. **(h)** The relationship between TMB and PTPRC expression level. **(i)** The relationship between TMB and PTPRC mutation.

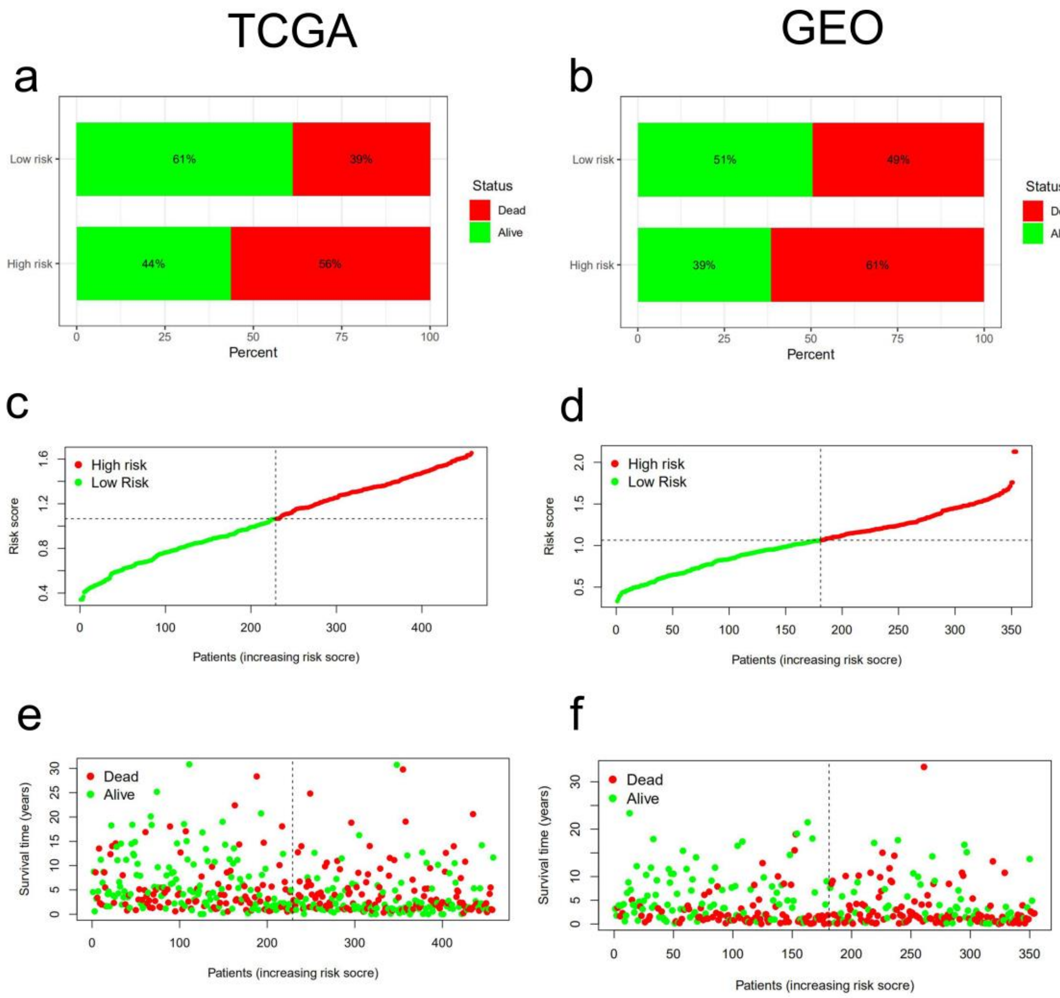

FigS3. The RiskScore distribution, survival status, and survival time between two clusters.

(a, c, e) The distribution of survival status, RiskScore, and survival time in training cohort**.**

(b, d, f) The distribution of survival status, RiskScore, and survival time in testing

cohort**.**

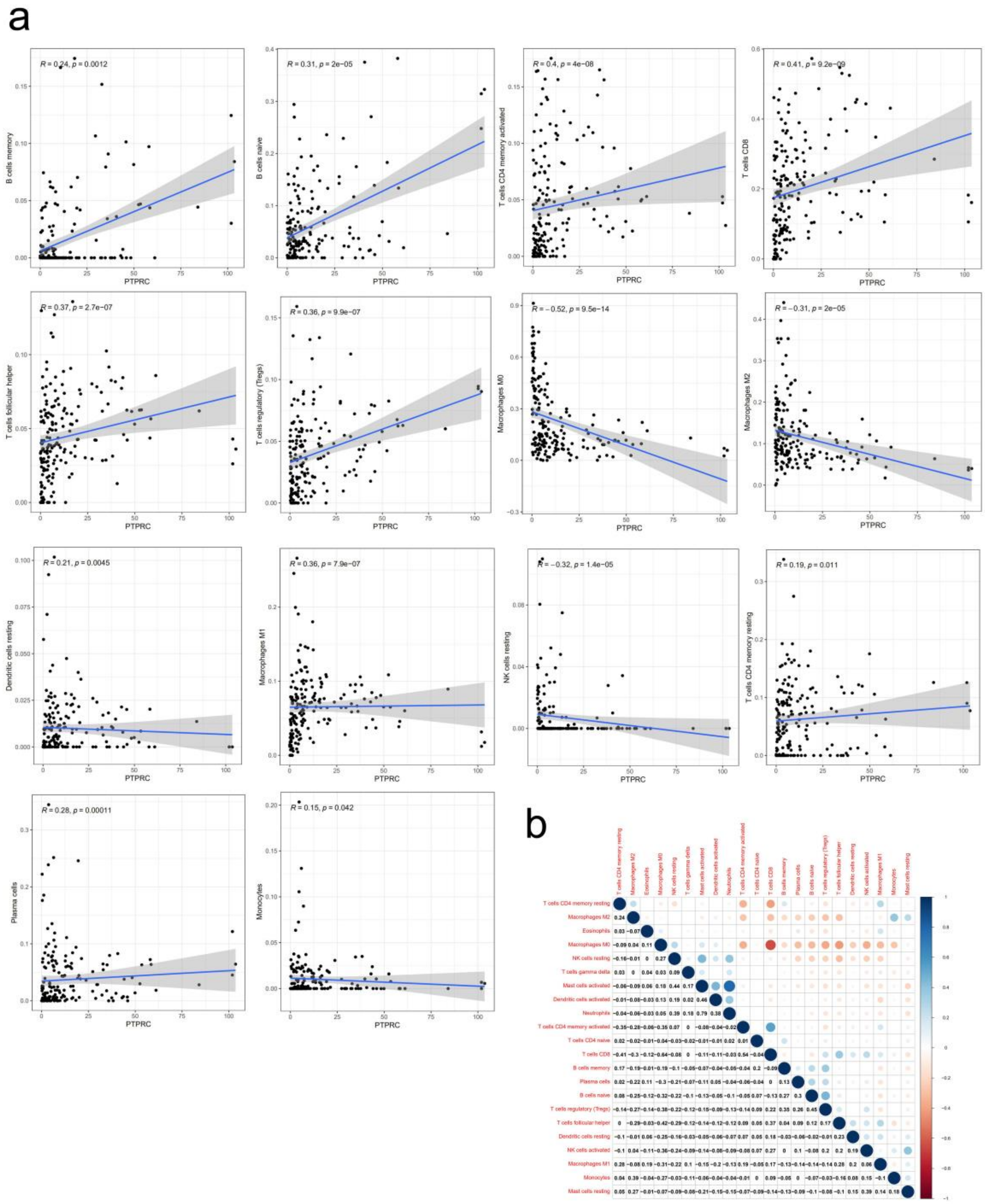

Fig S4. The relationship between PTPRC and immune cell infiltration.

(**a**) Correlation analysis of the relationship between PTPRC expression and immune cell infiltration.

(**b)** The correlation between different immune cells. The red represents positive correlation, and the blue represents negative correlation.

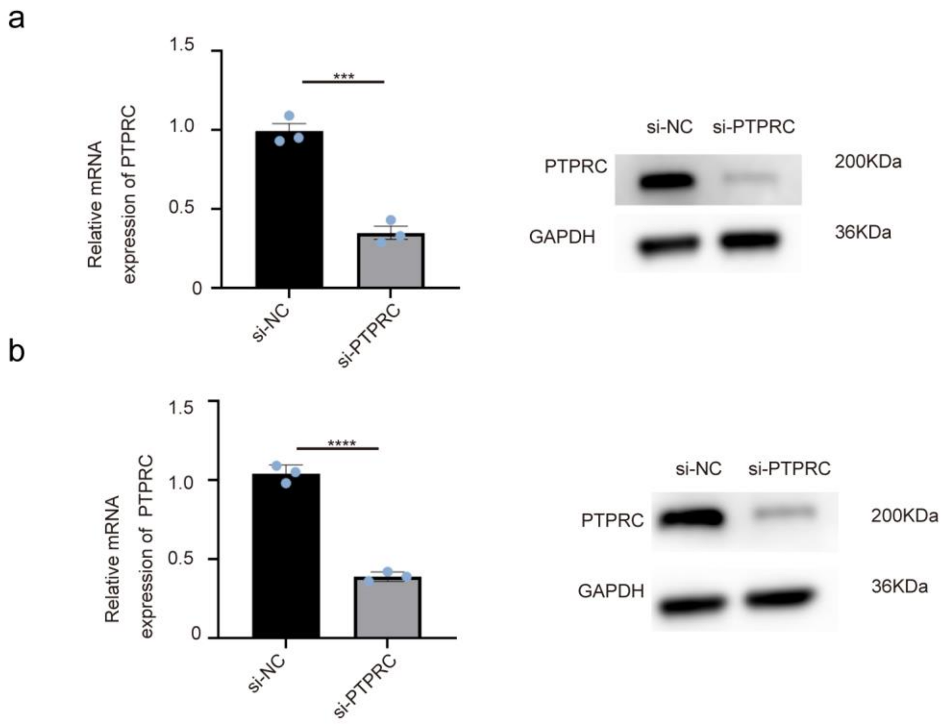

Fig S5. Knockdown of PTPRC in A375 and MEL-28 cells.

A375(a) and MEL-28 (b) cells were transfected with si-NC or si-PTPRC and then subjected to a Western blot analysis (the figures have been cropped) and qPCR, GAPDH were used as loading controls, respectively.

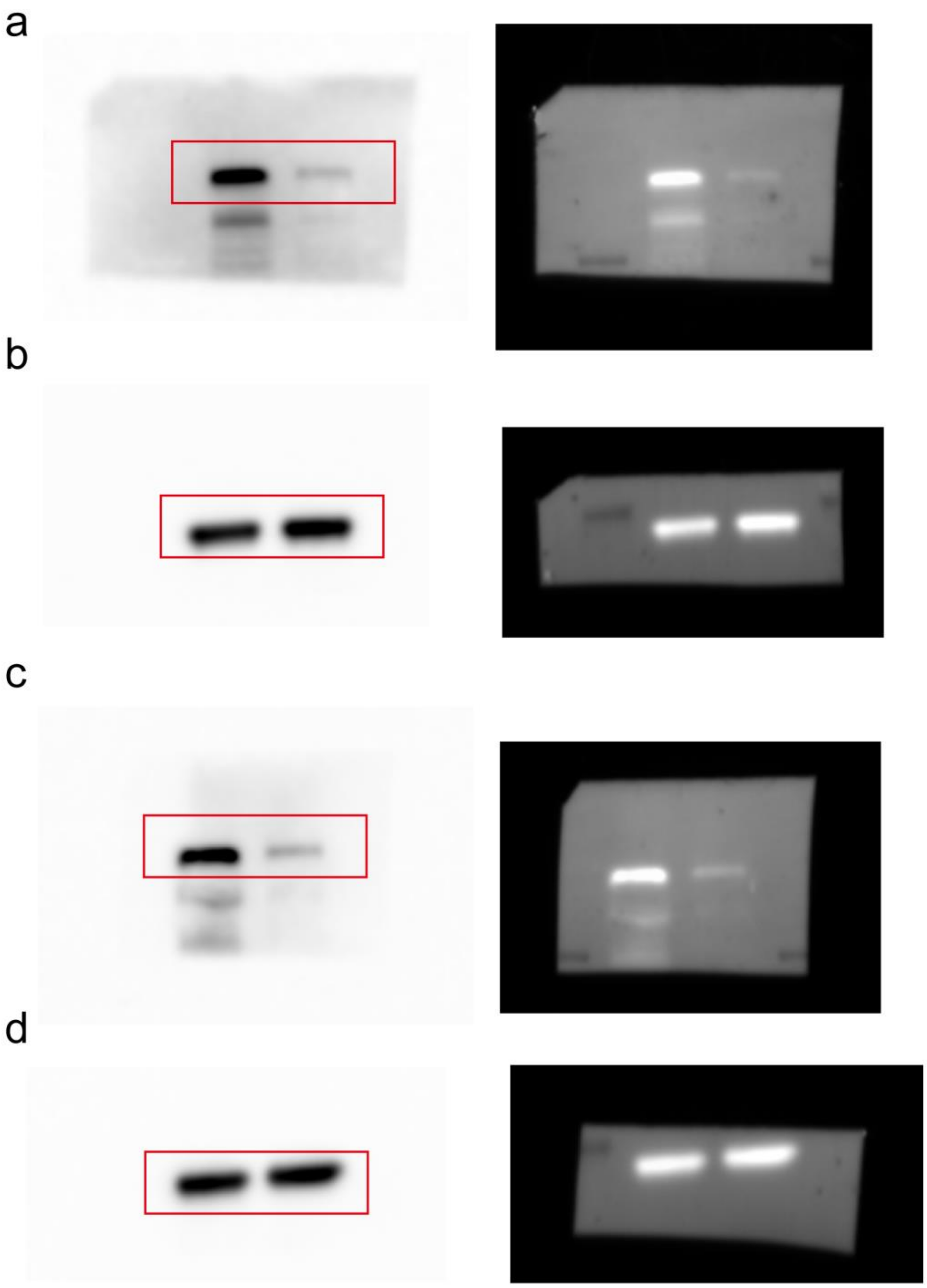

FigS6. Original western blots in the paper

Protein expression of PTPRC (a) and internal reference protein (b) in si-NC and si-PTPRC in A375 cells. Protein expression of PTPRC (c) and internal reference protein (d) in si-NC and si-PTPRC in MEL-28 cells. The red box shows the cropped section. The reason our membranes are smaller is that during development, we crop extraneous strips to save antibodies.
